# Is Social Network Diversity Associated with Tooth Loss among Older Japanese Adults?

**DOI:** 10.1371/journal.pone.0159970

**Published:** 2016-07-26

**Authors:** Jun Aida, Katsunori Kondo, Tatsuo Yamamoto, Masashige Saito, Kanade Ito, Kayo Suzuki, Ken Osaka, Ichiro Kawachi

**Affiliations:** 1 Department of International and Community Oral Health, Tohoku University Graduate School of Dentistry, Sendai, Japan; 2 Center for Preventive Medical Sciences, Chiba University, Chiba, Japan; 3 Center for Gerontology and Social Science, National Center for Geriatrics and Gerontology, Obu, Japan; 4 Division of Dental Sociology, Department of Oral Science, Graduate School of Dentistry, Kanagawa Dental University, Yokosuka, Japan; 5 Department of Social Welfare, Nihon Fukushi University, Mihama, Japan; 6 Division of Oral Health Sciences, Department of Health Sciences, School of Health and Social Services, Saitama Prefectural University, Koshigaya, Japan; 7 Department of Policy Studies, Aichi Gakuin University, Nisshin, Japan; 8 Department of Social and Behavioral Sciences, Harvard T.H. Chan School of Public Health, Boston, Massachusetts, United States of America; Taipei Veterans General Hospital, TAIWAN

## Abstract

**Background:**

We sought to examine social network diversity as a potential determinant of oral health, considering size and contact frequency of the social network and oral health behaviors.

**Methods:**

Our cross-sectional study was based on data from the 2010 Japan Gerontological Evaluation Study. Data from 19,756 community-dwelling individuals aged 65 years or older were analyzed. We inquired about diversity of friendships based on seven types of friends. Ordered logistic regression models were developed to determine the association between the diversity of social networks and number of teeth (categorized as ≥20, 10–19, 1–9, and 0).

**Results:**

Of the participants, 54.1% were women (mean age, 73.9 years; standard deviation, 6.2). The proportion of respondents with ≥20 teeth was 34.1%. After adjusting for age, sex, socioeconomic status (income, education, and occupation), marital status, health status (diabetes and mental health), and size and contact frequency of the social network, an increase in the diversity of social networks was significantly associated with having more teeth (odds ratio = 1.08; 95% confidence interval, 1.04–1.11). Even adjusted for oral health behaviors (smoking, curative/preventive dental care access, use of dental floss/fluoride toothpaste), significant association was still observed (odds ratio = 1.05 (95% confidence interval, 1.02–1.08)).

**Conclusion:**

Social connectedness among people from diverse backgrounds may increase information channels and promote the diffusion of oral health behaviors and prevent tooth loss.

## Introduction

Severe tooth loss is the 36th most prevalent health condition in the world [1]. Tooth loss is common in the older population and exacerbates poor nutritional status in the elderly [2]. Being underweight is strongly associated with higher mortality—sometimes rather than being overweight—among Asian populations, including Japan [3,4]. Japan is an aging society with 25.1% of the population aged 65 or older in 2013, and this percentage is estimated to increase to 39.9% by 2060 [5]; the average life expectancy was 86.4 years for women and 79.9 years for men in 2012. Hence, tooth loss is an important public health problem in this aging society.

Social determinants of oral health inequalities have drawn increasing attention, including inequalities in tooth loss, which are the consequence of dental health behavior, diseases, and care throughout the life course of an individual [6,7,8,9]. Social integration is an important social determinant of behavior as it provide opportunities for sharing information, adopting norms of behavior, and supporting individuals’ decision making [10].

Previous studies have reported that larger social networks are associated with oral health [11,12,13,14,15]. However, despite previous reports on social networks and oral health, the mechanism for this association remains unclear, because most of the studies only considered the size of the network. In the present study, we hypothesized that social network diversity is associated with tooth loss.

## Materials and Methods

### Study design, participants and setting

The present cross-sectional study was based on data from the Japan Gerontological Evaluation Study (JAGES) project. The JAGES project is an ongoing prospective cohort study investigating social and behavioral factors associated with health among individuals aged 65 years or older [6,9,16,17].

Between August 2010 and January 2012, self-administered questionnaires were mailed to 169,215 community-dwelling older individuals in 31 municipalities throughout Japan, and 112,123 individuals responded (response rate = 66.3%). Participants in each municipality were randomly selected from among community-dwelling people aged 65 or older who were free of physical or cognitive disability i.e. individuals not currently receiving public long-term care insurance benefits. Approximately one-fifth of the total cohort were randomly selected to receive a supplemental survey inquiring about oral health behaviors. 23,050 individuals responded to the supplemental survey. Respondents with missing data about their number of remaining teeth and social network variables were excluded; hence, data from 19,756 respondents were analyzed.

### Dependent variable

The number of remaining teeth was determined by self-report. Participants were asked how many remaining teeth they have and the number was grouped into one of the following four categories: ≥20 teeth, 10–19 teeth, 1–9 teeth, or no teeth. This categorical variable was used as the dependent variable in ordered logistic regression analysis.

### Independent variables

We created a social network diversity variable based on questionnaire responses about the types of friends often met: 1) people living in the same area (neighbors), 2) childhood friends, 3) friends from school days (school friends), 4) current or former work colleagues (colleagues), 5) friends sharing the same interests or leisure activities, 6) friends involved in the same volunteer activities, and 7) other. The responses were summed and analyzed as a continuous variable ranging from 0 to 7. We also assessed social network size by asking about the number of friends/acquaintances seen over the past month, categorized as follows: none, 1 to 2, 3 to 5, 6 to 9, and ≥10. Lastly, we asked about social network contact frequency by the following question: “How often do you see your friends?” The choices were as follows: 1) Almost everyday, 2) twice or three times a week, 3) once a week, 4) once or twice a month, 5) several times a year, and 6) rarely.

For sociodemographic covariates, we included sex, age group, educational attainment, equivalized household income, longest job, and marital status. For health-related covariates that could be associated with oral health and social networks, diabetes history [18,19] and depressive symptoms [20,21] (15-item Geriatric Depression Scale [22]: low risk [0–4 points], medium risk [5–9 points], or severe risk [10–15 points]) were considered.

For the oral health behavior variables we asked about smoking status, history of curative dental visits, history of preventive dental visits, interdental brushing/dental floss use, and fluoride toothpaste use. Smoking status was categorized as never smoked, quit 5 years or earlier, quit 4 years or later, and current smoker. Dental visits for treatment/prevention were ascertained by asking, “Did you visit a dentist for treatment/prevention in the last 6 months?” We also asked about whether respondents used interdental brush/dental floss and offered three choices: no, sometimes, and everyday. Fluoride toothpaste use was classified as yes, unsure about fluoride but use toothpaste, and no.

### Analysis

Ordered logistic regression models were developed to estimate the odds ratio (OR) and 95% confidence intervals (95% CI) for having different numbers of remaining teeth. First, to estimate the independent associations of each social network variable, we separately examined the association of each social network variable with adjustment for sociodemographic and health covariates. In the second step, all three social network variables were included simultaneously in the same model together with sociodemographic and health covariates. Finally, oral health behavior variables are added to the second model. To check the robustness of the results, sensitivity analyses were conducted in which all social network variables were treated as continuous variables. When analyzing the data, missing responses regarding sociodemographic covariates and oral health variables were included as the dummy variable. We used the Stata 13.1 software package (Stata Corp LP, College Station, Texas, US) for statistical analysis.

### Ethical considerations

The questionnaire and an explanation of the study were sent to the participants by mail. The participants were informed that participation was voluntary and that returning the self-administered questionnaire would be interpreted as implying consent. Ethical approval for the study was obtained from the Ethics Committee at Nihon Fukushi University.

## Results

Among the 19,756 respondents, 53.3% were women. The mean age of the participants was 73.9 years (standard deviation = 6.1). The proportion of respondents with ≥20 teeth, 10–19 teeth, 1–9 teeth, and no teeth were 34.1%, 26.0%, 25.4%, and 14.5%, respectively. Table 1 shows the distribution of respondents by variable and number of remaining teeth. Respondents with higher network diversity, network size, and network contact frequency tended to have 20 or more teeth. The distribution of respondents with 20 or more teeth varies from 25.6% to more than 40% for network diversity variables, which seems larger compared to other network variables. Older respondents and those with lower socioeconomic status, bereavement, diabetes, depression, and poor dental behaviors tended to have a lower number of teeth.

**Table 1:** Distribution of respondents by variable and number of remaining teeth (N = 19,756).

		% of respondents	Number of remaining teeth (%)			
			≥20	10–19	1–9	0
Network diversity	0	3.3	25.6	23.3	29	22.2
(Number of type of friends)	1	44.6	28.6	25.6	27.7	18.1
	2	31.3	37.5	26.8	24.2	11.5
	3	14.8	41.6	26.1	22.4	9.9
	4	4.5	42.9	26.7	20.4	9.9
	5	1.2	43.5	26.2	19.8	10.5
	6	0.3	44.2	21.2	23.1	11.5
	7	0.0	42.9	42.9	14.3	0
Network contact frequency	Rarely	7.2	23.6	25.1	29.3	22
(Frequency of meeting friends)	Several times a year	16.1	35.1	26.6	25	13.3
	1 or 2 times/month	20.9	34.8	26.5	25.2	13.5
	Once a week	17.9	36.2	26.7	23.6	13.5
	2 or 3 times/week	23.6	35.2	25.5	25.5	13.8
	Almost everyday	14.4	32.8	25.2	26.3	15.6
Network size	0	5.2	27.1	25.4	28	19.4
(Number of friends met in a month)	1–2	17.3	29.6	25.5	28.1	16.7
	3–5	26.4	31.1	25.4	27.5	16
	6–9	14.0	34.6	27.9	24.9	12.5
	>9	37.1	39.1	26.1	22.4	12.3
Age	65–69	30.1	46.4	29.4	18.9	5.3
	70–74	29.2	37.4	27.8	25.1	9.7
	75–79	21.8	28.2	24.6	29.6	17.6
	80–84	12.7	19.6	21.4	31.2	27.9
	≥85	6.2	9.3	16.2	32.1	42.4
Sex	Men	46.7	35.1	25.9	24.7	14.4
	Women	53.3	33.3	26.2	26.1	14.5
Equivalized income	Lowest	40.9	30.3	26	27.9	15.8
	Middle	33.5	40.6	27.1	21.7	10.5
	Highest	9.3	44.1	25.2	19.2	11.5
Education	<6 years	2.2	10.2	19.2	33.1	37.5
	6–9 years	43.3	28.2	25.6	28.2	17.9
	10–12 years	35.2	37.8	27.4	23.8	11
	>12 years	17.5	45.3	25.9	20.1	8.7
Longest job	Professional and technical	14.8	42.6	27.1	21	9.4
	Administrative	5.6	42.7	27.4	20.3	9.5
	Clerical	14.1	43.5	28.8	19.7	8
	Sales and service	13.3	33.4	27.7	27.8	11.1
	Skilled and labor	13.0	33.6	25.5	26.9	14
	Agriculture, forestry and fishery	7.7	17.3	22.3	31	29.4
	Others	12.1	29.7	24.7	27.8	17.8
	No occupation	5.3	29.9	24	26.3	19.8
Marital status	Married	71.8	37.5	26.5	23.9	12.1
	Bereaved	21.7	24.3	23.9	29.7	22.2
	Divorced	3.1	28.9	27.4	30.5	13.2
	Never married	1.8	37	31.2	21.4	10.3
Diabetes	Yes	63.9	33.8	26.1	25.2	14.9
	No	12.7	29.9	25.5	27.9	16.7
Depressive symptoms	None	60.2	37.6	26	23.5	13
	Mild depression	17.7	28.5	26.2	28.3	17.1
	Severe depression	5.9	23.2	24.7	32.8	19.3
Smoking	Never smoked	54.8	36.4	26.3	24.3	13
	Quit 5 years or earlier	21.3	37.6	25	23.3	14.2
	Quit 4 years or later	4.7	26.8	28.4	28.8	16
	Current smoker	10.1	24.1	27.1	31	17.9
Curative dental visit in last 6 months	Yes	48.0	33.5	30.7	28.4	7.5
	No	45.5	35.1	21.8	22.2	20.9
Preventive dental visit in last 6 months	Yes	26.1	38.2	28.3	26.7	6.7
	No	54.3	32.1	24.9	24.7	18.2
Interdental brush/dental floss use	Everyday	20.8	43.8	28.2	21.5	6.6
	Sometimes	17.6	40.4	31.9	23	4.7
	No	40.6	26.4	23	29.2	21.4
Fluoride toothpaste use	Yes	35.3	36.4	29.7	24.6	9.3
	Use any toothpaste	26.7	35.6	27.6	27.1	9.7
	No	16.7	26.4	17.9	26.1	29.7

**Fig 1:**
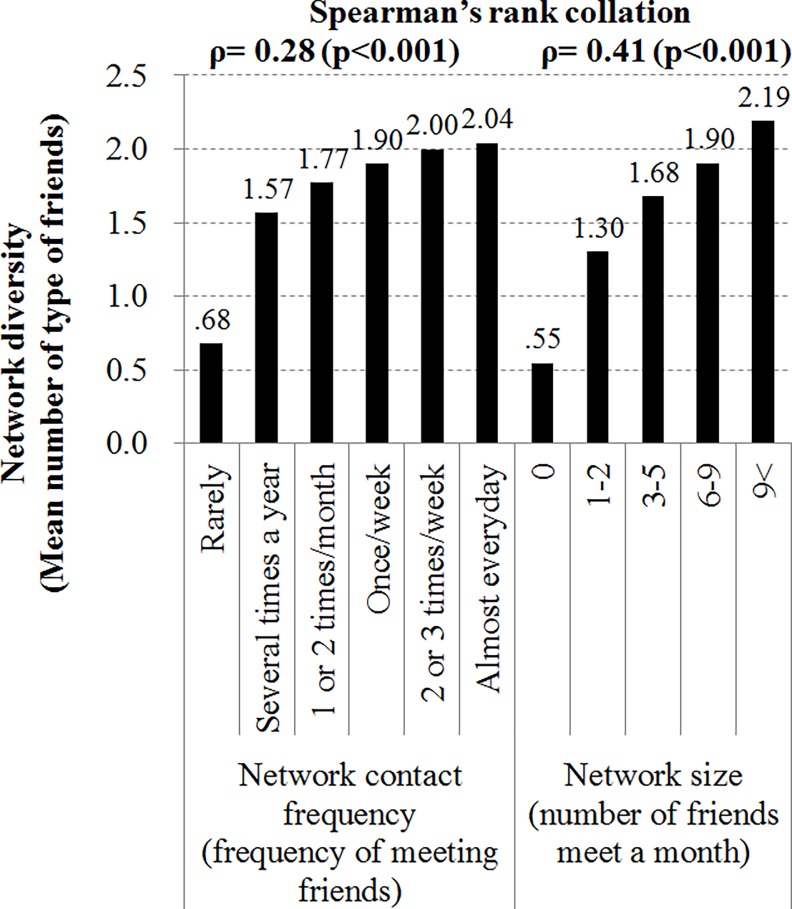
Mean network diversity (number of type of friend) by social network size and contact frequency.

Fig 1 shows the mean network diversity (different types of friendships) according to social network size and contact frequency. There were significant correlations between the network contact frequency and size. Spearman’s rank correlation between network diversity and network size was 0.41 (p<0.001), and between network diversity and network contact frequency was 0.28 (p<0.001).

Table 2 shows the results of multivariate ordered logistic regression analysis. After adjusting for sociodemographic and health covariates, social network variables were significantly associated with a higher number of remaining teeth. Each unit increment in the diversity of social networks was significantly associated with having more teeth (OR = 1.09; 95% CI, 1.07–1.12). After including all three social network variables in the same model, the odds ratios of the network variables became decreased. For network diversity, the odds ratio decreased slightly to 1.08 (95% CI, 1.04–1.11). The odds ratios for network size were no longer statistically significant. In the final model, after adjusted for oral health behavior variables, the odds ratio of network diversity was further decreased to 1.05 (95% CI, 1.02–1.08).

**Table 2:** Odds ratios (OR) and 95% confidence intervals (CIs) of variables for retaining a large number of teeth (N = 19,756).

		Separately included models*^1^				Simultaneously included model*^2^				Oral health behaviors adjusted *^3^			
		OR	95%CI		p-value	OR	95%CI		p-value	OR	95%CI		p-value
			Low	High			Low	High			Low	High	
Network diversity	(number of type of friends)	1.09	1.07	1.12	<0.001	1.08	1.04	1.11	<0.001	1.05	1.02	1.08	0.002
Network contact frequency (Frequency of meeting friends)	Rarely	1.00				1.00				1.00			
	Almost everyday	1.20	1.06	1.35	0.003	1.09	0.95	1.26	0.231	1.05	0.91	1.22	0.481
	2 or 3 times/week	1.36	1.21	1.52	<0.001	1.26	1.10	1.44	0.001	1.20	1.04	1.37	0.011
	Once/week	1.40	1.25	1.58	<0.001	1.33	1.16	1.53	<0.001	1.28	1.11	1.47	0.001
	1 or 2 times/month	1.33	1.19	1.48	<0.001	1.29	1.12	1.47	<0.001	1.22	1.07	1.40	0.003
	Several times a year	1.32	1.18	1.48	<0.001	1.29	1.14	1.47	<0.001	1.25	1.10	1.43	0.001
Network size (Number of friends meet a month)	0	1.00				1.00				1.00			
	1–2	1.12	0.99	1.28	0.074	0.93	0.80	1.07	0.321	0.93	0.80	1.08	0.336
	3–5	1.09	0.97	1.24	0.153	0.87	0.75	1.01	0.069	0.85	0.73	0.98	0.029
	6–9	1.24	1.08	1.41	0.002	0.97	0.83	1.14	0.738	0.94	0.80	1.10	0.439
	9<	1.25	1.11	1.41	0.000	0.99	0.85	1.16	0.928	0.96	0.82	1.12	0.630
Age	65–69					1.00				1.00			
	70–74					0.70	0.65	0.75	<0.001	0.65	0.61	0.70	<0.001
	75–79					0.46	0.42	0.49	<0.001	0.43	0.39	0.46	<0.001
	80–84					0.29	0.26	0.31	<0.001	0.27	0.25	0.30	<0.001
	85-					0.15	0.13	0.17	<0.001	0.15	0.13	0.17	<0.001
Sex	Men					1.00				1.00			
	Women					1.10	1.04	1.17	0.001	0.81	0.75	0.88	<0.001
Equivalent income	Lowest					0.71	0.64	0.78	<0.001	0.73	0.66	0.80	<0.001
	Middle					0.92	0.83	1.01	0.089	0.94	0.85	1.04	0.208
	Highest					1.00				1.00			
Education	<6 years					0.49	0.40	0.59	<0.001	0.52	0.43	0.64	<0.001
	6–9 years					0.67	0.62	0.72	<0.001	0.67	0.62	0.73	<0.001
	10–12 years					0.81	0.75	0.88	<0.001	0.82	0.76	0.89	<0.001
	>12 years					1.00				1.00			
Longest job	Professional and technical					1.00				1.00			
	Administrative					0.97	0.85	1.11	0.691	0.97	0.85	1.11	0.700
	Clerical					1.03	0.93	1.14	0.569	1.00	0.90	1.11	0.987
	Sales and service					0.75	0.68	0.83	<0.001	0.75	0.68	0.83	<0.001
	Skilled and labor					0.83	0.75	0.92	<0.001	0.83	0.75	0.92	<0.001
	Agriculture, forestry and fishery					0.41	0.36	0.46	<0.001	0.42	0.38	0.48	<0.001
	Others					0.72	0.65	0.80	<0.001	0.73	0.66	0.81	<0.001
	No occupation					0.78	0.68	0.89	<0.001	0.79	0.68	0.90	0.001
Marital status	Married					1.00				1.00			
	Bereaved					0.80	0.75	0.86	<0.001	0.84	0.78	0.90	<0.001
	Divorced					0.76	0.65	0.88	<0.001	0.86	0.74	1.00	0.047
	Never married					1.13	0.93	1.37	0.204	1.19	0.98	1.44	0.082
Diabetes	Yes					1.00				1.00			
	No					0.76	0.70	0.82	<0.001	0.78	0.72	0.85	<0.001
Depressive symptom	Normal					1.00				1.00			
	Mild					0.82	0.76	0.88	<0.001	0.85	0.79	0.91	<0.001
	Severe					0.71	0.64	0.80	<0.001	0.73	0.65	0.82	<0.001
Smoking	Never									1.00			
	Quitted 5 years or past									0.81	0.74	0.88	<0.001
	Quitted 4 years or later									0.52	0.45	0.59	<0.001
	Current									0.47	0.42	0.52	<0.001
Curative dental visit in this 6 month	Yes									1.00			
	No									1.04	0.98	1.11	0.238
Preventive dental visit in this 6 month	Yes									1.00			
	No									0.76	0.71	0.82	<0.001
Interdental brush/dental floss use	Every day									1.00			
	Sometime									0.91	0.84	0.99	0.027
	No									0.52	0.48	0.56	<0.001
Fluoride toothpaste use	Yes									1.00			
	Use any toothpaste									1.02	0.96	1.09	0.539
	No									0.51	0.47	0.55	<0.001

*1: Each social network variable was included separately in the model. Models adjusted for age, sex, income, education, job, marital status, diabetes, and mental health.

*2: Social network variables were included simultaneously with adjustment for the aforementioned sociodemographic and health covariates.

*3: In addition to social network variables and sociodemographic and health covariates, smoking, curative dental visits, preventive dental visits, interdental brush/dental floss use, and fluoride toothpaste use were added to the model.

In the sensitivity analyses, all social network variables were treated as continuous variables (Table 3). The odds ratios of network diversity in these analyses were similar to those the previous analyses in Table 2.

**Table 3:** Odds ratios (OR) and 95% confidence intervals (CIs) of social network variables, treated as continuous variables, for retaining a large number of teeth (N = 19,756).

	Separate models*^1^				Simultaneous model*^2^				Adjusted for oral health behaviors*^3^			
	OR	95%CI		p-value	OR	95%CI		p-value	OR	95%CI		p-value
		Low	High			Low	High			Low	High	
Network diversity (number of type of friends)	1.09	1.07	1.12	<0.001	1.08	1.05	1.12	<0.001	1.06	1.02	1.09	<0.001
Network contact frequency (Frequency of meeting friends)	1.01	1.00	1.03	0.119	0.99	0.97	1.01	0.154	0.98	0.96	1.00	0.078
Network size (Number of friends meet a month)	1.05	1.03	1.07	<0.001	1.04	1.01	1.06	0.006	1.03	1.00	1.05	0.048

*1: Each social network variable was included separately in the model. Models adjusted for age, sex, income, education, job, marital status, diabetes, and mental health.

*2: Social network variables were included simultaneously with adjustment for the aforementioned sociodemographic and health covariates.

*3: In addition to social network variables and sociodemographic and health covariates, smoking, curative dental visits, preventive dental visits, interdental brush/dental floss use, and fluoride toothpaste use were added to the model.

## Discussion

To our knowledge, this is the first study to report the association of social network diversity on oral health. Our results suggest that social network diversity is associated with the number of remaining teeth independently of network size and contact frequency. Network diversity may mediate the association between the size of the social network and oral health. The significant association of network diversity suggests the importance of a social network with people from different social backgrounds, which may help the diffusion of information about oral health-promoting behaviors as well as the maintenance of healthy oral norms

Previous studies also reported the importance of social network diversity on health behavior and health. Kroenke et al. [23] examined the association between network diversity and various health behaviors including cancer treatment among women diagnosed with cancer. After accounting for network size, lower network diversity was significantly associated with health-compromising behaviors such as lack of exercise, obesity, smoking, and excessive alcohol intake. Moore et al. [24] reported the importance of social network diversity for smoking cessation. Association with higher diversity and physical activity was also reported [25]. In relation to oral health among older Japanese adults, diversity [26] and types of social participation [27] were associated with tooth loss. In contrast, Rafnsson et al. [28] reported that network diversity was not independently associated with subjective well-being, while network size and contact frequency showed significant association. For mental health outcomes, social network diversity did not seem to be independently associated [29,30].

The discrepancies among study results may be explained by differences in the mechanisms through which social integration affects health. Specifically, social network diversity may be helpful in diffusing information across social ties, i.e. having friends across diverse domains of life—neighbors, colleagues at work, friends in hobby groups—increases the probability that the ego will be exposed to new types of (health-relevant) information. On the other hand, network diversity may not be as useful in buffering psychological stress because the social ties might not be sufficiently intimate in order for the ego to look to these relationships for emotional support. In health behavioral pathways, obtaining new information and different behavioral norms from people from different social backgrounds is important [24,25,31,32]. The importance of network diversity is captured by the concept of bridging social capital, i.e., “*resources that are accessed by individuals as a result of their membership of a diverse network or a group*” [31]. Bridging social capital consists of the resources (e.g. information) obtained from the weak ties that link people from diverse backgrounds. Bridging social capital is one form of social capital and which is considered to be more effective in terms of providing various channels for the transfer of information and social influence among people of different social backgrounds [33,34]. In this respect, further longitudinal study is needed to examine the mediating effect of oral health behaviors on the relations of social network diversity and oral health. In addition, in our final model, social network variables still showed significant association even when adjusted for oral health behaviors. This association may be due to psychological stress-related pathways because stress is a risk of periodontal disease [35,36], an important cause of tooth loss as well as dental caries [37]. In addition to health behavior itself, norms within social groups are also believed to affect health in different aspects. For example, people may feel embarrassed about the appearance of their teeth compared to their neighbors’ [38]. Therefore, the good oral health of neighbors could improve one’s own oral health behaviors.

Previous public health interventions based on the social network and social capital concept have provided opportunities for social participation and promotion of health in older people [39,40,41]. These interventions potentially improve the social participation and communication of people with different social backgrounds. Ohura et al. [42] showed that a community intervention program increased opportunities for social participation, resulting in improvements in the exchange of health information. Further studies are needed to determine the possible impacts of similar interventions on oral health. In addition, since there are differences in oral health between urban and rural areas, targeting high-risk populations especially in under-served rural areas would be useful.

Depression is considered to be a risk of poor oral health behavior, dental caries, and tooth loss [43,44]. In addition, a bi-directional relationship between social network and depression is suggested [45,46]. Therefore, we considered the depressive symptom variable as the covariate. In this study, depressive symptom was associated with lower number of remaining teeth. As this study is cross-sectional, further longitudinal studies to reveal the relationships among oral health, social network, and mental illness are required.

One limitation of this study was that we used self-reported questionnaires to obtain our data. However, the validity of self-reports of the number of teeth has been established previously [47]. Our use of categorical variables to group the remaining number of teeth (as opposed to using the exact number of teeth) also minimized the potential for information bias. The generalizability of the present results is also limited. Because the municipalities that participated in the study were not randomly selected and sampling weight was not applied, the present results cannot be applied to the entire Japanese population. As sociodemographic factors, such as population density and urban–rural definition, differed among countries, the generalizability of this study for other countries is also limited. In addition, we could not consider other oral health behaviors relating to social network such as unhealthy diet relating to caries and excess alcohol use and dental injury. This may lead to overestimating the effect of social network. There were other possibilities that we could not fully consider as potential confounders. For example, in relation to the network contact frequency variable, its association with teeth outcome seems not to be dose-dependent; the “Once a week” category showed the highest odds ratio (OR = 1.40) compared to the “Almost every day” category (OR = 1.20). One possible reason is that the communities in which people meet friends every day are relatively rural, and some community characteristics in such areas might affect oral health even if we adjusted for various factors including access to dental care. Finally, because of the cross-sectional design, the present study cannot claim causality. There is a possibility of bidirectional relationships between oral health and reduced social network. Longitudinal studies or experimental studies are needed for causal inference. The main strength of our study was that we used a large sample size and involved populations from various areas of Japan, thus allowing us to grasp differences in characteristics among neighborhoods and to improve the external validity of the study result in the Japanese population.

## Conclusion

Our study suggests that regardless of the strength of social networks, social connectedness among people from diverse backgrounds may increase information channels and promote the diffusion of oral health behaviors, thereby preventing tooth loss.
